# Mix Design of Pervious Concrete in Geotechnical Engineering Applications

**DOI:** 10.3390/ma18091909

**Published:** 2025-04-23

**Authors:** Maurizio Ziccarelli

**Affiliations:** Engineering Department, University of Palermo, Viale delle Scienze Edificio 8, 90128 Palermo, Italy; maurizio.ziccarelli@unipa.it

**Keywords:** permeable concrete, geotechnical applications, clogging, filter properties, residual permeability, durability

## Abstract

This paper presents a comprehensive experimental study on the mix design and performance of permeable concrete for geotechnical applications, focusing on its hydraulic conductivity, durability, and filter properties. Characterized by high porosity and minimal or no fine aggregates, classical pervious concretes are effectively utilized in various civil and environmental engineering applications, including drainage systems and erosion control. This research examines the influence of the particle size distribution of aggregates on the filter properties of permeable concrete for applications in geotechnical engineering (draining piles, deep trench drains, and draining backfill). It emphasizes the importance of resistance to clogging to maintain adequate residual hydraulic conductivity and to prevent the internal erosion of soils into which permeable concrete drains are installed. The experimental results indicate that including sand in the aggregates strongly enhances the filtering capacity of pervious concrete. These findings suggest that if the mix design of permeable concrete is developed considering the grain size distribution of the base soils, the concrete will meet long-term drainage requirements (sufficient residual hydraulic conductivity), exhibit good resistance to physical clogging, provide excellent protection for the base soils against internal erosion, and contribute to the overall stability of geotechnical systems.

## 1. Introduction

**Figure 1:**
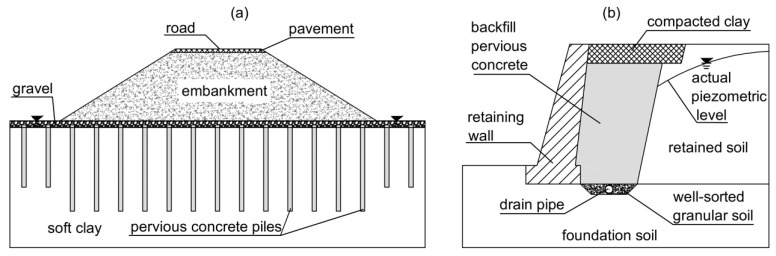
Example of pervious concrete used for geotechnical applications. (**a**) Embankment on saturated soft clay. The pervious piles accelerate the consolidation processes linked to the construction of the embankment and the application of service loads. (**b**) Backfill of permeable concrete utilized for the construction of a retaining wall.

**Figure 2:**
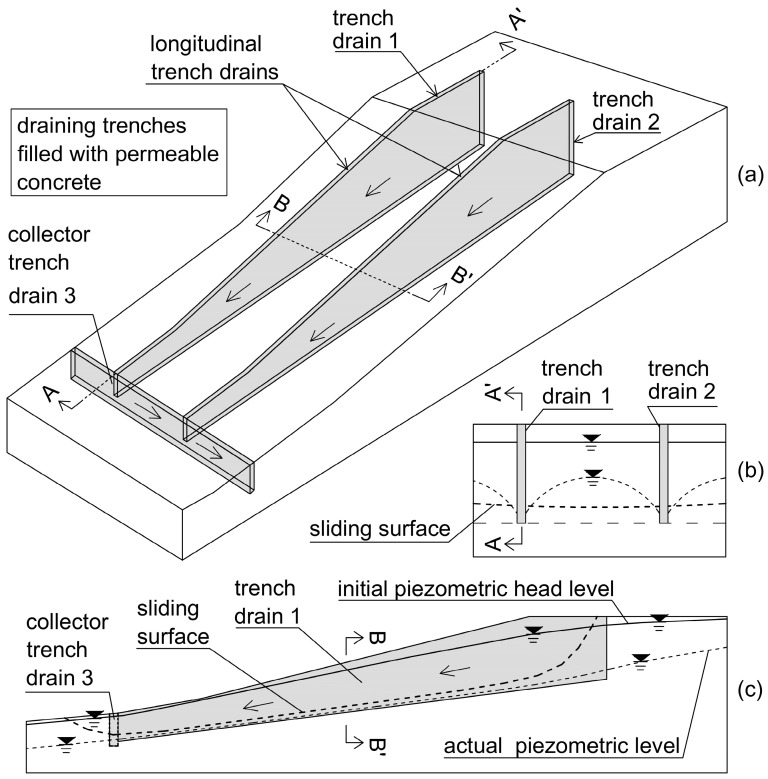
Example of the use of pervious concrete for geotechnical applications. (**a**) Three-dimensional schematic representation of draining trenches (realized with permeable concrete) employed to enhance the stability conditions of marginally stable slopes or for the stabilization of landslides; (**b**) vertical transverse cross-section B-B′; and (**c**) vertical longitudinal section A-A′ (along trench drain 1). Redrawn from Ziccarelli and Valore, 2019 [37].

Permeable concrete (also known as enhanced-porosity, porous, pervious, or voided concrete) is employed as both a lightweight structural material and a non-structural one in applications related to civil and environmental engineering. It is used for pavement roads, residential streets, greenhouses, parking lots and bathing areas, footpaths, sports courts, reef blocks, porous pipes, decorative pavements, noise barriers, etc. [1,2,3,4,5,6,7]. Porous concrete is even used to mitigate heat conditions in urban islands [8,9,10,11] and decrease heavy metals in runoff through a complicated process connected to the variability of the dimensions, distribution, and winding nature of voids [12,13,14,15,16]. Additionally, due to the relatively low costs of its primary materials (cement, aggregates, and water) and its very simple preparation, pervious concrete is an economical and relatively environmentally sustainable material [17,18,19,20,21,22]. The permeable concrete employed for the applications mentioned above is “no-fines concrete”, that is, concrete that contains no sand or only a minimal amount. It is characterized by a significant number of pores, normally varying between 15% and 40%. Recently, permeable concrete was suggested for applications in geotechnics, i.e., developments on soft ground, to reduce the risk of liquefaction in highly loose, saturated sand deposits [23,24,25,26,27,28,29,30,31,32,33,34]; as draining backfill for gravity walls; and for the construction of deep trench drains [35,36,37,38,39], as shown in Figure 1 and Figure 2. Recently, permeable concrete was also suggested as a protective material to combat erosion on deep slopes, incorporating sustainable vegetation practices [40,41,42,43,44].

Many experimental studies have been conducted on permeable concrete for draining road pavement, as well as various applications in environmental and civil engineering e.g., [7,8,11,45,46]. For these applications, the pervious concrete must have a permeability adequately elevated to “capture” and quickly discharge whatever rainwater has dropped onto the paving area within the below layers to prevent aquaplaning. It must also gather this contaminated water, preventing it from penetrating the ground beneath. For these applications, it is not necessary for pervious concrete to operate as a filter, although it has outstanding filtration qualities for heavy metals and chemical compounds. In cases of clogging, the pavement can be treated to recover the initial permeability, for example, through rejuvenation treatments [47,48,49,50].

In the geotechnical problems represented in Figure 1a, where permeable concrete can be used very effectively, the principal purpose of piles is to accelerate the processes of consolidation for constructions on saturated soft soils, reducing the excess of interstitial pressures and supporting the loads transmitted to the soil and piles by the superstructure. This accelerates the settlements associated with the increase in effective stresses. When pervious concrete is utilized as a draining backfill for walls, as shown in Figure 1b, the main purpose is to control the interstitial pressures and thus reduce the thrust to which the wall is subjected, consequently increasing the overall stability of the wall.

When permeable concrete is utilized for deep draining trenches (Figure 2), the principal purpose is to permanently reduce the interstitial pressures in the soil mass where the trench drains are installed. For these applications, in different soils, particularly fine-grained ones, the processes are linked to the relatively reduced flow velocity of the water conveyed toward the piles, the backfill of the walls, and the draining trenches. For these applications, permeable concrete must possess effective filtration properties and, therefore, the ability to safeguard the soils in which it is embedded to prevent internal erosion.

The key feature of permeable concrete for geotechnical applications is its permeability. This should be sufficiently higher than the hydraulic conductivity of the surrounding soil to favor the flow of groundwater toward backfill, trench drains, or piles and to subsequently direct the drained water to a discharge location or for final delivery. Of course, considering the crucial aims of reducing pore water pressures (for draining trenches), controlling interstitial pressure (as refill behind retaining walls), and reducing the excess of interstitial pressures on piles, pervious concrete must also function as a filter material to prevent the internal erosion of the base soils. Additionally, it should have high resistance to clogging (primarily physical clogging), which can impede its draining capacity. In other words, its “residual” permeability should be adequately elevated to guarantee the primary purpose of the intervention. Crucially, the rejuvenation treatments described for road pavements do not apply to permeable concretes used in geotechnical applications.

Previous studies [35,37] have demonstrated that it is possible to develop mix designs that concurrently fulfill multiple requirements. Some of these concretes have been extensively investigated through laboratory tests. To this end, Ziccarelli and co-workers [37,38,39] suggested a mix design for pervious concrete containing a comparatively large amount of sand that meets all the aforementioned criteria at once. It is widely recognized that the hydraulic conductivity of permeable concrete (as well as any granular material) relies on several factors, including overall porosity, the size and arrangement of pores, pore connectivity, and (especially) constrictions. The factors mentioned above are all closely interrelated and influenced by other factors related to the characteristics of pervious concrete. The most significant of these are the water–cement ratio, the aggregate–cement ratio, the cement content, the energy of compaction, the curing environment, and the grain size distribution of the aggregates. In piles and deep trench drains, the vertical effective stress resulting from the self-weight of fresh concrete plays a crucial role, particularly concerning deeper layers. Given that the depth of piles or trenches can reach 40 m or more, the stress associated with the self-weight during the casting and curing phases of fresh concrete can be significantly higher than the typical stress levels observed in laboratory conditions, which only account for the self-weight of the sample. This elevated stress can impact the properties of pervious concrete, including its hydraulic conductivity in its final state or after the hardening phase.

To the best of the author’s knowledge, no exhaustive research has been conducted on the filtering characteristics that permeable concrete must possess for geotechnical purposes, particularly deep draining trenches. This study presents and discusses the results of a comprehensive experimental investigation into the mix design of permeable concrete aimed at achieving good filtering properties, adequate residual hydraulic conductivity, and high durability in relation to the granulometric composition of underlying soils.

## 2. Principal Criteria of Permeable Concrete for Geotechnical Applications

The main requirements of pervious concrete for applications in geotechnical engineering (see Figure 1 and Figure 2) are as follows.

### 2.1. Permeability

A very important feature of permeable concrete as a draining material is its hydraulic conductivity. It must be adequately higher than the permeability of the base soil (the soil surrounding piles or trench drains and retained soil located behind a wall) to favor groundwater flow toward piles, trench drains, or backfill. In the first case, the flow of the water toward piles accelerates the consolidation processes, i.e., dissipating the interstitial pressures in excess, to accelerate the transfer of loads transmitted by superstructures to piles and foundation soils and, hence, to accelerate settlement. In the second and third cases, the drained water must be conveyed to exit points or drains. In these last two cases, of course, the vital and crucial purpose of drainage is the permanent reduction in pore water pressures to reduce the thrust in the case of walls and improve the overall stability of the walls, for the enhancement of the stability conditions of slightly stable slopes, and for the stabilization of landslides in slopes with high interstitial pressures [51,52,53,54,55,56,57,58,59,60,61,62,63].

Moreover, since the deep strata of pervious concrete utilized for deep trench drains or draining piles are subjected to high stress levels as a result of the self-weight of cast-in-place fresh concrete, the permeability of these strata must be sufficient for the scope of the design. In other terms, considering that the vertical stress of the fresh concrete cast in place is linked linearly to the depth of the considering strata, the design values of the permeability must be considered in relation to the deep strata. The hydraulic conductivity of very deep strata can reduce to 50% of that relative to strata subjected to very low stress levels [40].

### 2.2. Stability of the Internal Structure

Permeable concrete is characterized by internal stability, as its structure consists of cemented particles that cannot migrate or move from one point to another within the volume of the system. Consequently, permeable concrete is not susceptible to internal erosion or piping flow erosion processes, which can occur in granular cohesionless soils [64].

### 2.3. Resistance to Clogging and Filtering Capacity

The permeable concrete used for geotechnical applications must have long-term “design hydraulic conductivity”; in other words, it must satisfy draining purposes for the entire existence of a geotechnical system. For this, it must have high resistance to clogging (primarily physical clogging) or sufficient residual hydraulic conductivity to guarantee its draining capacity. Furthermore, it must possess filter properties to avoid internal erosion of the soil where piles or draining trenches are installed as well as the retained backfill soils (base soils). This peculiarity is of fundamental importance both to avoid erosion of the base soil and hence problems of deformations, settlements, distortions, etc., of the superstructure and to reduce the clogging that is linked to the flow of fine soil particles into it.

### 2.4. Strength and Stiffness

**Figure 3:**
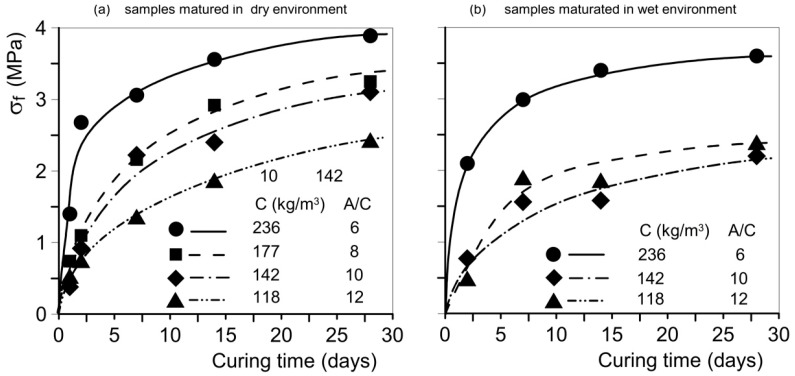
Trend of compressive strength in the function of curing time for different aggregate–cement (A/C) ratios, cement content (C), and environment of maturation: (**a**) dry, (**b**) wet. Redrawn from [37].

Pervious concrete must have significant long-term strength and stiffness to guarantee the aims for which it is employed. In particular, it must have enough resistance to safely transfer vertical loads to depth strata for piles and trench drains and sufficient stiffness to reduce deformations, settlements, and distortions in superstructures. Moreover, it must have sufficient strength after a few days of its casting to facilitate the quick construction of contiguous trench drain panels. This rapid strength development is essential for maintaining an organized construction site and controlling costs [37], as it allows for the efficient sequencing of construction activities. Furthermore, the elevated shear, compressive resistance, and stiffness of permeable concrete allow it to function as a “shear key” in cases where draining trenches are constructed parallel to the sliding direction, provided that the trench drains are adequately embedded below the sliding surface in stiff and stable layers. These types of trench drains, called “counterfort drains”, can significantly contribute to increasing overall slope stability. This is because the shear resistance of permeable concrete is greater than what is present on the slip surface due to soil alone [39]. In cases in which pervious concrete is employed as backfill material behind retaining walls, it can normally provide sufficient stiffness and strength, and no further requirements are necessary. The findings reported in [35,37,39] have demonstrated that pervious concrete possesses all the strength (Figure 3) and stiffness characteristics described above.

### 2.5. Cast in Place

Due to system geometry, the problem of casting in place is not significant for the backfill of walls because normally the depths of the concrete placed are small. To prevent segregation in trench drains and draining piles, permeable concrete should be poured in situ using the well-known tremie pipe technique [65]. Segregation can be minimized by selecting a relatively uniform aggregate grade, a low water–cement ratio, and low cement content.

## 3. Materials

Permeable concrete is a composite material made with aggregates (such as gravel and sand), cement, water, and occasionally additives. The following sections outline the key properties of the materials used for confectioning the pervious concrete for the experimental study. The characteristics of aggregates and clogging materials have been determined according to ASTM standards [66,67,68].

### 3.1. Aggregates

**Figure 4:**
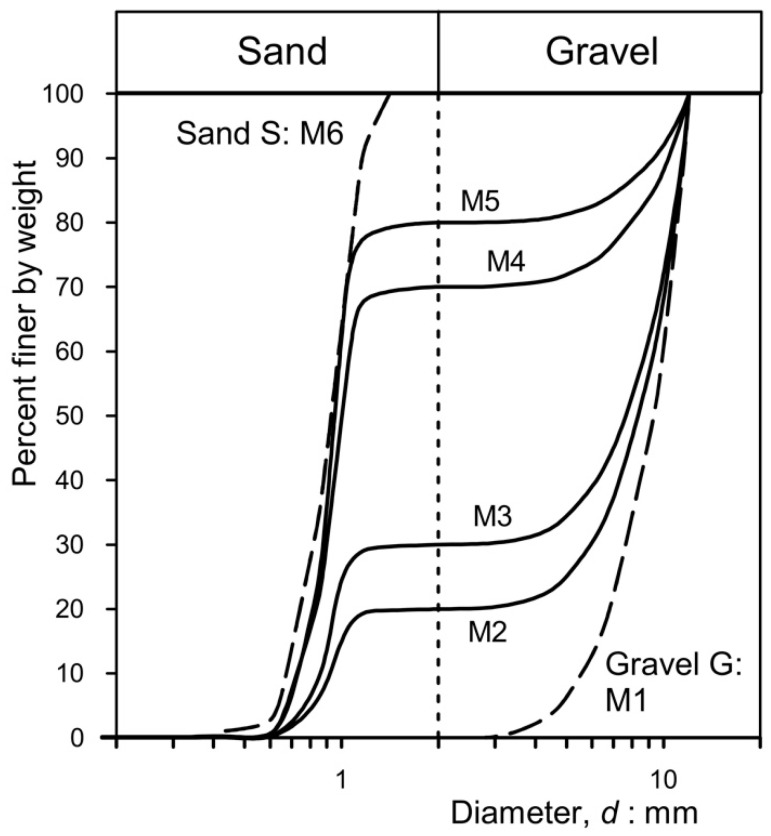
Initial grading of the aggregates used to prepare the permeable concretes.

Fine gravel, G (M1), and coarse to medium sand, S (M6), were used (Figure 3) as aggregates to mix the pervious concrete samples used in this experiment. The characteristics of sand S and gravel G are summarized in Table 1. Gravel G and sand S are uniformly graded; the sand grains are equidimensional and subrounded and consist of quartz, while the gravel particles are angular or subangular and consist of whitish limestone. Figure 4 shows the grading of mixtures M2-M5, obtained using a combination (by weight) of sand S and gravel G, as summarized in Table 2. Mixtures M2-M5 are gap-graded. The coefficient of uniformity, CU, of gravel G (M1) is 1.41, that of sand S (M6) is 1.78, while the CU of mixtures M2-M5 varies from 1.36 for M5 to 10.36 for M3.

### 3.2. Cement

The properties of the Portland cement used are as follows: Tecnocem II B-LL 32.5 R (EN 197-1 [69]—Cem II/B-LL 32.5). The composition, by mass, of the cement is as follows: (a) clinker between 65% and 79%; (b) limestone between 21% and 35%; (c) TOC (total organic carbon) ≤ 0.20%; and (d) minor constituents (chlorides, sulfates such as SO_3_, etc.) ≤ 0.10%.

### 3.3. Water

Tap water from the Palermo aqueduct was used. The principal characteristics of the water are summarized in Table 3.

### 3.4. Clogging Materials

**Figure 5:**
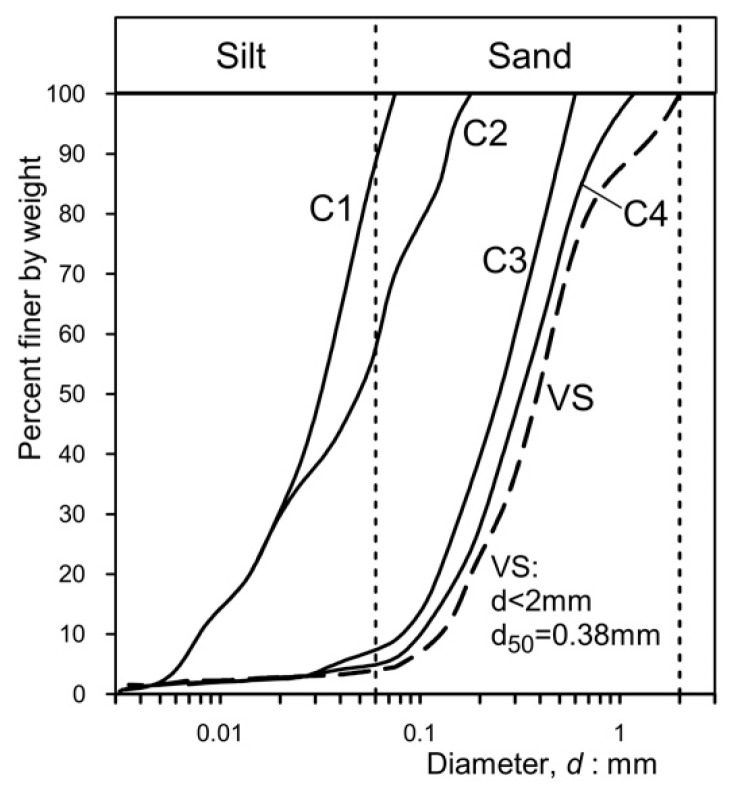
Granulometric distribution of the clogging materials used in the experiments. Materials C1–C4 were obtained by sieving natural volcanic sand (VS).

To examine the filter properties and assess the residual permeability (an evaluation of effectiveness over time or durability) of the permeable concretes utilized in the experiments, a series of falling head permeability tests were conducted on concrete specimens that had previously undergone a series of “clogging cycles” until residual hydraulic conductivity, if present, was achieved. The grain size distribution of the “clogging materials” employed in the tests is depicted in Figure 5.

Fine sand and silt were utilized. Clogging materials C1–C4 were derived by pulverizing dark gray volcanic sand (VS, from the Aeolian Islands); its grading is also illustrated in Figure 5.

The principal characteristics of the clogging materials are presented in Table 4. These materials have been chosen because they are cohesionless and non-plastic and, hence, easy to utilize and represent the most vulnerable soils with respect to internal erosion phenomena.

## 4. Methods

All mixtures (M1–M6) used in the experimental program were prepared with W/C = 0.4 (water–cement ratio), C = 142 kg/m^3^ (cement content), and A/C = 10 (aggregate–cement ratio). The values of W/C, A/C, and C represent an optimum mix design to ensure that the pervious concrete achieves sufficient strength and stiffness a few days after casting, good hydraulic conductivity, and adequate workability [35,36,37,38].

### 4.1. Tests of Permeability

**Figure 6:**
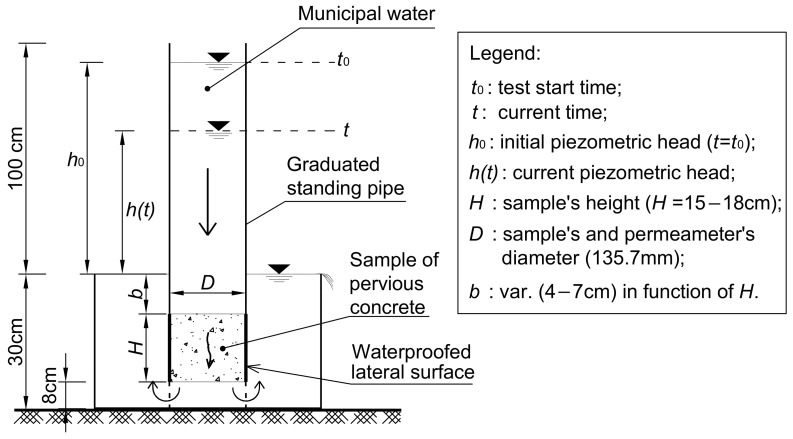
Permeameter scheme (falling head) employed to carry out the hydraulic conductivity tests. The perspex graduated standpipe stands on three 8 cm long legs. To ensure 1D vertical flow, the sample surface was waterproofed with polyurethane resin. For other details, see [35].

The hydraulic conductivity, *k*, of the pervious concrete specimens was determined by employing a variable head permeameter (Figure 6). The specifics of the test methodology are outlined in [35]. The tests were conducted in a laboratory at 20 °C ± 1 °C. This type of test (variable head permeameter) is widely employed by researchers to determine the permeability of porous concrete [70,71,72]. The piezometric gradient, *i*, interval ranged from 0.1 to 1, corresponding to the range of practical interest in geotechnical engineering works, e.g., draining piles, deep trench drains, and retaining-wall backfills. The coefficient of permeability, *k*, was evaluated using the following equation (see Figure 6):

(1) $$k=\frac{A_{1}H}{A_{2}\left(t-t_{0}\right)}\ln(\frac{h_{0}}{h_{t}})$$

where

- A_1_ and A_2_ are the cross-sectional areas of the sample and the graduated pipe of the permeameter, respectively (A_1_ = A_2_ = πD^2^/4);
- H is the length of the porous concrete sample;
- h_0_ is the initial piezometric head, and h_t_ is the current piezometric head at times t_0_ and t, respectively.

This relationship holds true under the hypotheses of Darcy’s law and laminar flow. In 2006, Montes and Haselbach [73] showed that the flow in pervious concrete lies within the laminar regime (or, at the very most, the transitional one).

Therefore, the above equation applies to the tested pervious concretes, as the mean dimensions of the aggregate’s particles—and, consequently, the dimensions of channels (voids and constrictions) in the employed permeable concrete—are smaller than those of the pores and constrictions in no-fines concrete.

### 4.2. Clogging Tests

**Figure 7:**
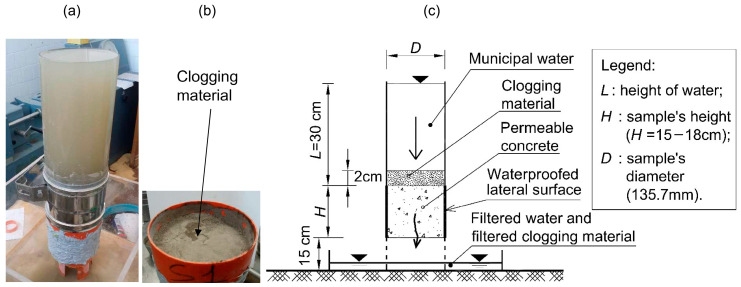
(**a**) Photo of a clogging test (at the start of the test) and head of the sample with the placed clogging material, measuring 2 cm thick (**b**). (**c**) Scheme of the test (similar to the falling head permeameter). The length of the samples (H) varies between 15 and 18 cm; D = 135.7 mm.

The mix design of the permeable concretes for geotechnical applications capable of simultaneously satisfying the draining and filtering requirements, related to the granulometric composition of the base soil, was studied through clogging tests and permeability tests. Figure 7 shows a photograph taken at the beginning of the clogging test, depicting the top of the sample. A 2 cm thick layer of clogging material was cast in place by gently tamping before the test started. The figure also depicts a schematic representation of the test setup. Clogging tests are similar to permeability tests, with the key difference being the presence of a clogging material layer at the top of the sample. Naturally, these tests are not indicative of the permeability of the permeable concrete, as the overall permeability is governed by the permeability of the combined system formed by the layer of clogging material and the porous concrete sample. During the test, particles from the clogging material are transported by seepage forces through the permeable concrete sample. Some particles become trapped within “constriction” voids after traveling a short distance through the concrete, while others exit through the bottom face of the specimen.

**Figure 8:**
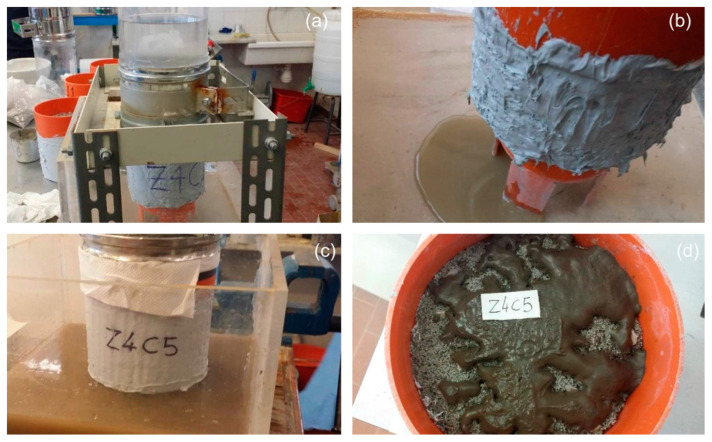
(**a**) The clogging test conducted on the sample made of mixture M5 (the water level is visible at about halfway through the test). (**b**) Water containing clogging material in the initial phase of the test. (**c**) Water containing clogging material after the experiment. (**d**) The sample’s head at the end of the test. Only a part of the initial clogging material remains at the head of the sample.

Figure 8 shows four photos illustrating the main phases of the clogging test. The sample consists of the M5 mixture, comprising 20% gravel (G) and 80% sand (S) by aggregate weight.

At the end of the test, the following materials were identified: A, the material remaining at the top of the sample; B, the material that passed through the sample; and P, the material retained inside the sample. The quantities of A and B were collected and weighed after being oven-dried, while P was determined by subtracting these values from the initial dry weight of the clogging material (W = 140 g).

This amount of clogging material is among the highest reported in the literature for clogging tests conducted on permeable concrete used in road pavements [74,75,76,77,78,79,80,81,82,83,84].

After each clogging cycle, a permeability test was conducted with a hydraulic gradient *i* ranging from 1 to 0.05, and the hydraulic conductivity was determined. For the first five clogging cycles, a clogging cycle and a permeability test were alternated. Subsequently, the permeability test was performed after every five clogging cycles.

## 5. Mechanisms of Clogging

**Figure 9:**
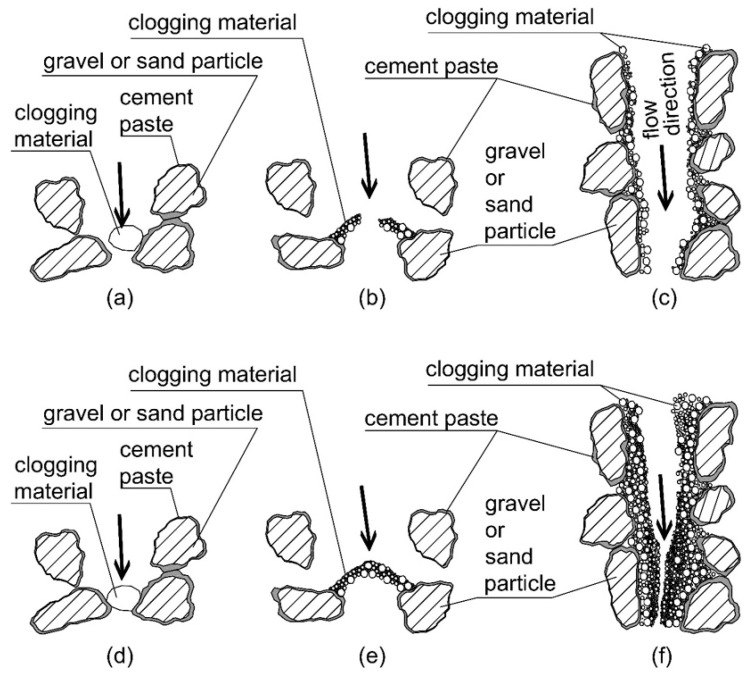
Mean mechanisms of physical clogging that can affect permeable concrete in geotechnical applications, with a simplified bidimensional representation of the evolution of the three types of clogging. (**a**,**d**) The total occlusion of a constriction caused by a relatively wide clogging material particle; (**b**,**e**) the occlusion caused by the growth of fine particles forming a bridge; and (**c**,**f**) the accumulation of fine particles on the “walls” of a large channel. The arrows indicate the direction of flow.

**Figure 10:**
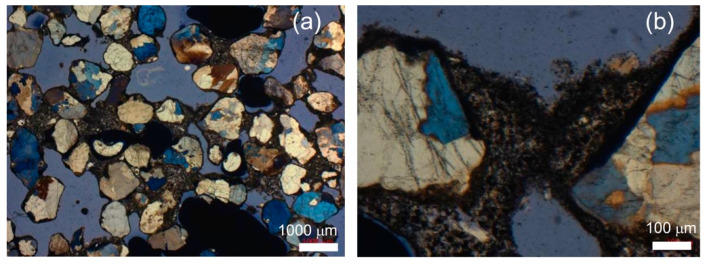
Microphotographs under cross-polarized light of thin sections of pervious concretes after clogging tests. (**a**) Pores completely filled with clogging material and particles surrounded by clogging material. (**b**) Mechanism of clogging caused by the growth of fine particles forming a bridge (mechanisms (b) and (e), Figure 9b,e).

In soils, clogging mechanisms can be classified as physical, chemical, or bacteriological. Among these, physical clogging is certainly the most relevant for the application of permeable concrete in geotechnical engineering. Figure 9 illustrates a model of the main physical clogging mechanisms. Naturally, the clogging processes depicted in Figure 9 are always present simultaneously and interdependently. Figure 10 shows two photos at different magnifications of thin sections observed under an optical microscope (Olympus model SZX12, equipped with IMAGE-PRO 6.3). To highlight the pores of the permeable concrete, a blue-colored resin was injected before preparing the thin section. Thin sections were obtained after clogging tests to observe the material deposited within the pores of the permeable concrete. The images reveal voids (in blue), gravel and sand particles, the clogging material, and the clogging mechanisms.

## 6. Results and Discussion

### 6.1. Hydraulic Conductivity

**Figure 11:**
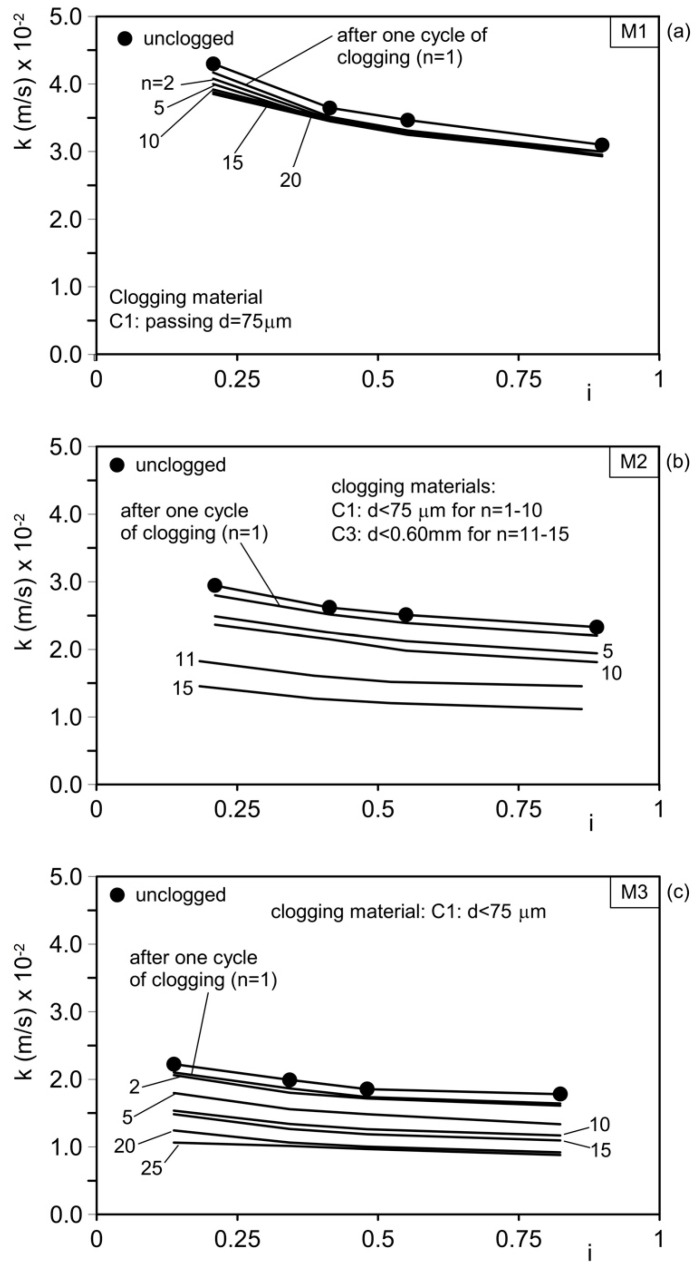
Results of permeability tests performed on unclogged material (solid line with filled circles) and after *n* cycles of clogging (lines without symbols). (**a**) Mix M1 (gravel G = 100%; sand S = 0); (**b**) mix M2 (gravel G = 80%; sand S = 20%); and (**c**) mix M3 (gravel G = 70%; sand S = 30%). *i*: hydraulic gradient.

**Figure 12:**
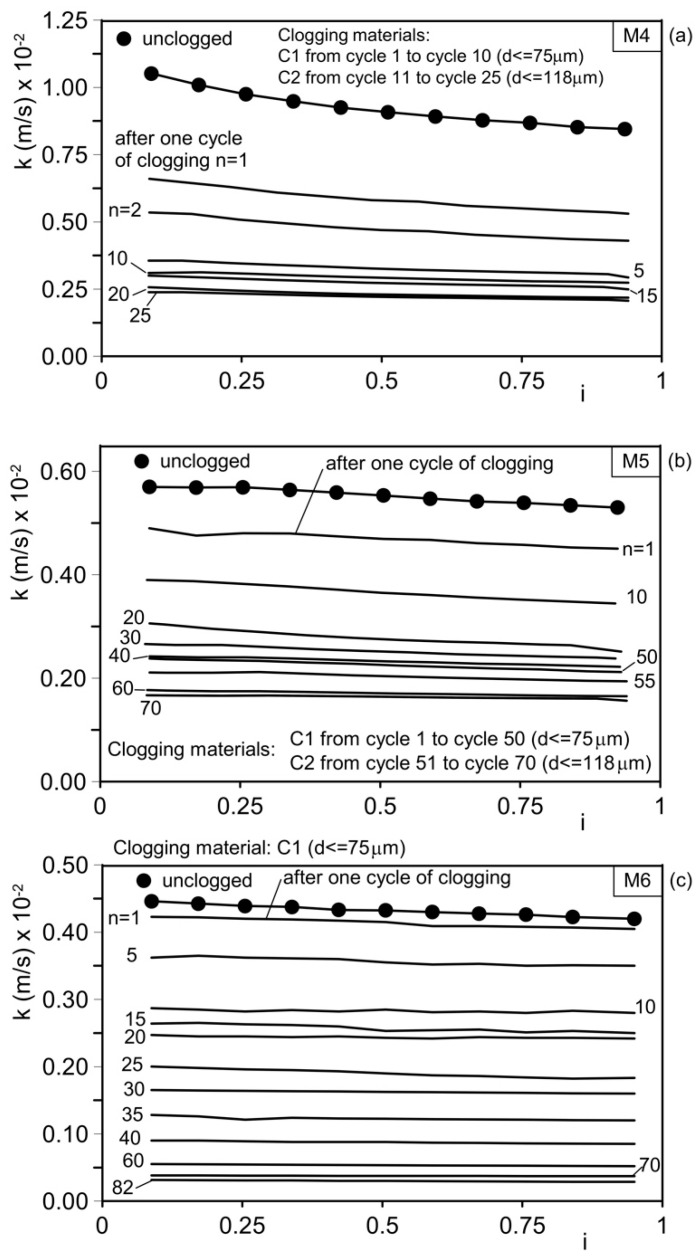
Results of permeability tests performed on unclogged material and after *n* cycles of clogging. (**a**) Mix M4 (gravel G = 30%, sand S = 70%); (**b**) mix M5 (gravel G = 20%, sand S = 80%); and (**c**) mix M6 (gravel G = 0, sand S = 100%). Note the different scales of hydraulic conductivity in the 3 diagrams. *i*: hydraulic gradient.

The results of the permeability tests conducted on the unclogged materials, as well as after *n* clogging cycles, are shown as a function of the clogging material used for mixtures M1–M3 and M4–M6 in Figure 11 and Figure 12, respectively. The hydraulic conductivity, *k*, exhibits slight variations with the hydraulic gradient, *i*. The initial (unclogged) hydraulic conductivity decreases as the mean aggregate diameter (d_50_) used to mix the pervious concrete decreases. Table 5 summarizes the results.

The initial hydraulic conductivity, *k*_ini1_, ranges from 4.3 × 10⁻^2^ m/s (4.3 cm/s) for permeable concrete M1 (aggregates composed entirely of gravel) to 0.45 × 10⁻^2^ m/s (0.45 cm/s) for permeable concrete M6 (aggregates composed entirely of sand). Mixtures M2–M5 show intermediate unclogged permeability values.

**Figure 13:**
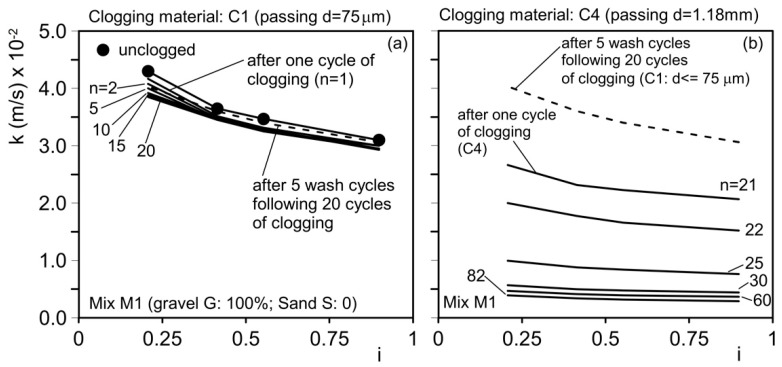
Results of permeability tests after n cycles of clogging. Mix M1 (gravel G = 100%; sand S = 0): (**a**) unclogged material and after first 20 clogging cycles with clogging material C1; (**b**) 62 subsequent cycles with clogging material C4 after 5 washing cycles. Note that the hydraulic conductivity after the washing cycles is about the same as the unclogged value. *i*: hydraulic gradient.

Mixture M1 was initially subjected to 20 clogging cycles using clogging material C1 (particles with a diameter smaller than 75 µm), as shown in Figure 11a. After these 20 cycles, the hydraulic conductivity decreased to 3.85 × 10⁻^2^ m/s, corresponding to 89.5% of the initial value. A large portion of the clogging material passed through the sample because the voids and constrictions were larger than the clogging particles, resulting in only a minimal amount of material remaining in the sample. These results demonstrate that mixture M1 (aggregates composed solely of gravel) performs very well in terms of “residual hydraulic conductivity” but poorly in terms of “base soil protection against internal erosion”. The sample was then subjected to five washing cycles to remove as much of the deposited material as possible, followed by 62 additional clogging cycles using a larger clogging material (C4, d ≤ 1.18 mm), as shown in Figure 13. After the washing cycles, the hydraulic conductivity was 3.98 × 10⁻^2^ m/s, an intermediate value between the unclogged permeability and the value measured after 20 clogging cycles.

Based on these observations, different mixtures were tested under various clogging conditions, as summarized in Table 3 and illustrated in Figure 11 and Figure 12.

For mixture M1, after 62 clogging cycles with material C4, the hydraulic conductivity decreased to 0.47 × 10⁻^2^ m/s, about 11% of the initial value and 10% of the unclogged value. Although this permeability is still sufficient to ensure drainage performance, it is inadequate in protecting the base soil from internal erosion.

Mixture M2 was subjected to 10 clogging cycles with material C1, followed by 5 clogging cycles with material C3, as shown in Figure 11b. During the first five clogging cycles with the finer material (C1), the hydraulic conductivity decreased from 2.94 cm/s (2.94 × 10⁻^2^ m/s) to 2.3 cm/s (2.30 × 10⁻^2^ m/s), representing a 20% reduction. During the subsequent five clogging cycles with material C3, the hydraulic conductivity further decreased from 2.3 cm/s (2.30 × 10⁻^2^ m/s) to 1.38 cm/s (1.38 × 10⁻^2^ m/s), corresponding to a 40% reduction in just five cycles. Overall, the reduction in permeability compared with the initial unclogged value exceeded 50%.

**Figure 14:**
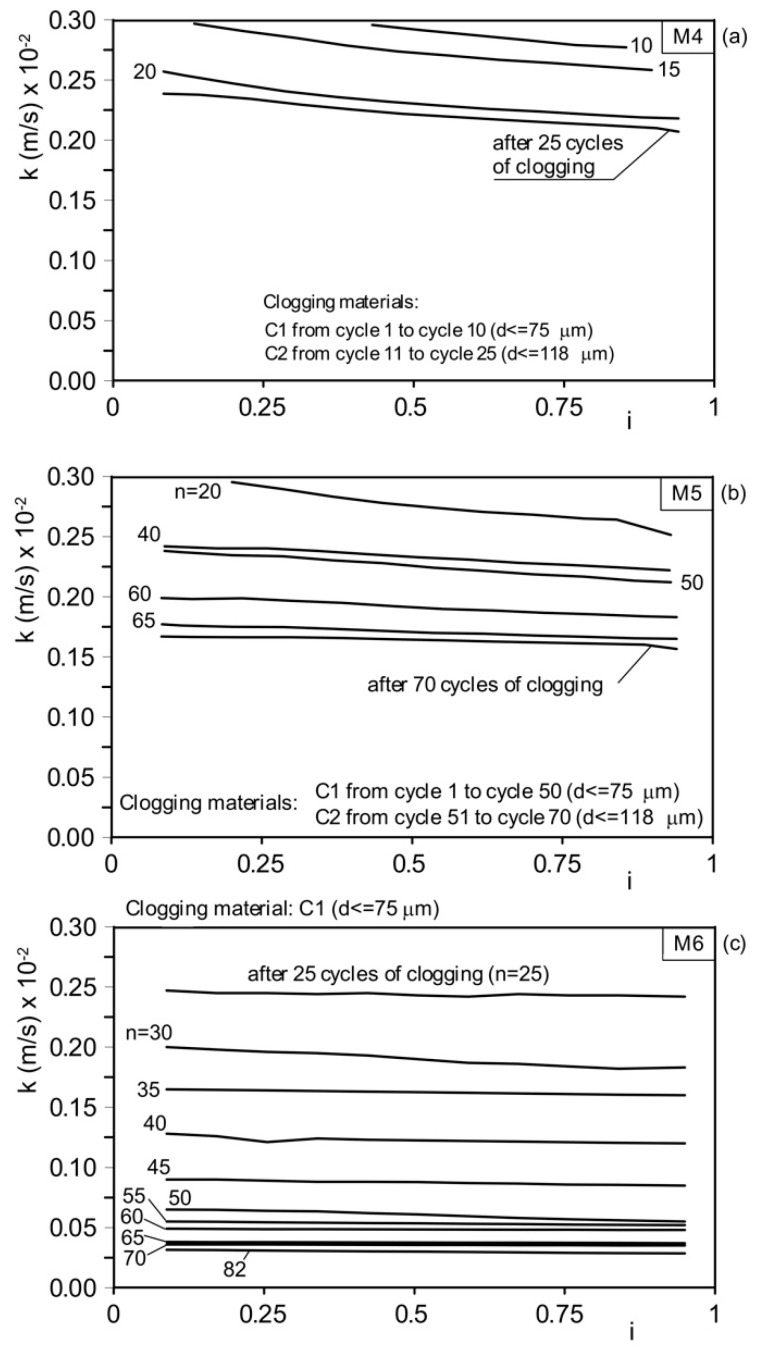
Results of permeability tests after *n* cycles of clogging. Magnification of the final parts of the results in Figure 12. (**a**) Mix M1 (gravel G = 100%; sand S = 0); (**b**) mix M2 (gravel G = 80%; sand S = 20%); and (**c**) mix M3 (gravel G = 70%; sand S = 30%). *i*: hydraulic gradient.

Mixture M3 (Figure 11c) was subjected to 25 clogging cycles using clogging material C1. As a result, the hydraulic conductivity decreased from 2.23 cm/s (2.23 × 10⁻^2^ m/s) to 1.01 cm/s (1.01 × 10⁻^2^ m/s), representing a 55% reduction. Mixture M4 was subjected to 10 clogging cycles with clogging material C1, followed by 15 clogging cycles with clogging material C2 (Figure 12a and Figure 14a). During the first 10 clogging cycles using the finer material (C1), the hydraulic conductivity decreased from 1.05 cm/s (1.05 × 10⁻^2^ m/s) to 0.31 cm/s (0.31 × 10⁻^2^ m/s), corresponding to a 70% reduction compared with the initial unclogged value. Over the next 15 clogging cycles (using material C2), the permeability coefficient further decreased from 0.31 cm/s (0.31 × 10⁻^2^ m/s) to 0.24 cm/s (0.24 × 10⁻^2^ m/s), indicating an additional 23% reduction. The total reduction in hydraulic conductivity, relative to the initial unclogged value, was approximately 77.1%.

Mixture M5 was subjected to 50 clogging cycles with clogging material C1, followed by 20 clogging cycles with clogging material C2 (Figure 12b and Figure 14b). During the first 50 clogging cycles, the permeability coefficient decreased from its original unclogged value of 0.57 cm/s (0.57 × 10⁻^2^ m/s) to 0.21 cm/s (0.21 × 10⁻^2^ m/s), representing a 63% reduction. Over the subsequent 20 clogging cycles, the hydraulic conductivity coefficient further decreased from 0.21 cm/s (0.21 × 10⁻^2^ m/s) to 0.17 cm/s (0.17 × 10⁻^2^ m/s), corresponding to an additional 19% reduction. The total reduction in hydraulic conductivity, relative to the initial unclogged value, was approximately 70%.

Mixture M6 (Figure 11c and Figure 14c) was subjected to 82 clogging cycles with clogging material C1. As a result, the hydraulic conductivity decreased from 0.45 cm/s (0.45 × 10⁻^2^ m/s) to 0.03 cm/s (0.03 × 10⁻^2^ m/s), representing a reduction greater than 90%.

The results demonstrate that hydraulic conductivity decreases with the number of clogging cycles and with increased clogging material particle dimensions.

This occurs because the clogging material progressively accumulates within the concrete sample, partially filling the voids and obstructing pores, constrictions, and channels through the clogging mechanisms illustrated in Figure 9.

These findings indicate that permeable concrete mix designs must be selected based on the granulometric composition of the base soil, which must be protected from internal erosion.

The base soil also influences the long-term (residual) hydraulic conductivity of the permeable concrete and, consequently, the drainage performance of the intervention (e.g., draining piles, trench drains, and drainage backfills), as well as its geotechnical purposes.

For fine-grained base soils (fine sand, silt, clay, and mixtures thereof), permeable concrete should be designed using aggregates containing sand. Including sand in the mix creates smaller pores, channels, and constrictions that help protect the base soil.

By contrast, pervious concrete made solely with coarse aggregates (gravel only) is suitable for protecting base soils comprising medium to coarse sands.

### 6.2. Study of the Trend of Remaining, Passing, and Retained Material, and a Model of Hydraulic Conductivity with Clogging Cycles

**Figure 15:**
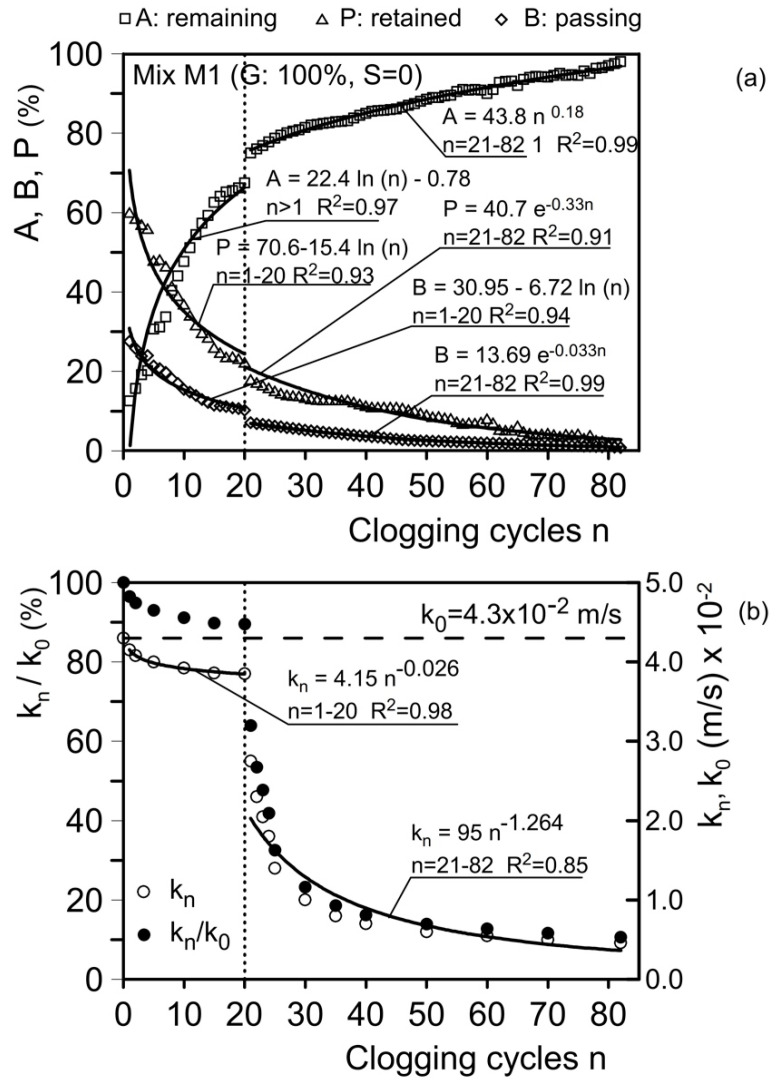
Results of clogging and permeability tests. (**a**) Material remaining at the head of the sample in weight (A), retained within the sample, (P) and passing through the sample (B); (**b**) hydraulic conductivity, *k*_n_, after *n* cycles of clogging and the *k*_n_/*k*_0_ ratio (*k*_0_: unclogged hydraulic conductivity). Mix M1 (gravel G = 100%; sand S = 0).

**Figure 16:**
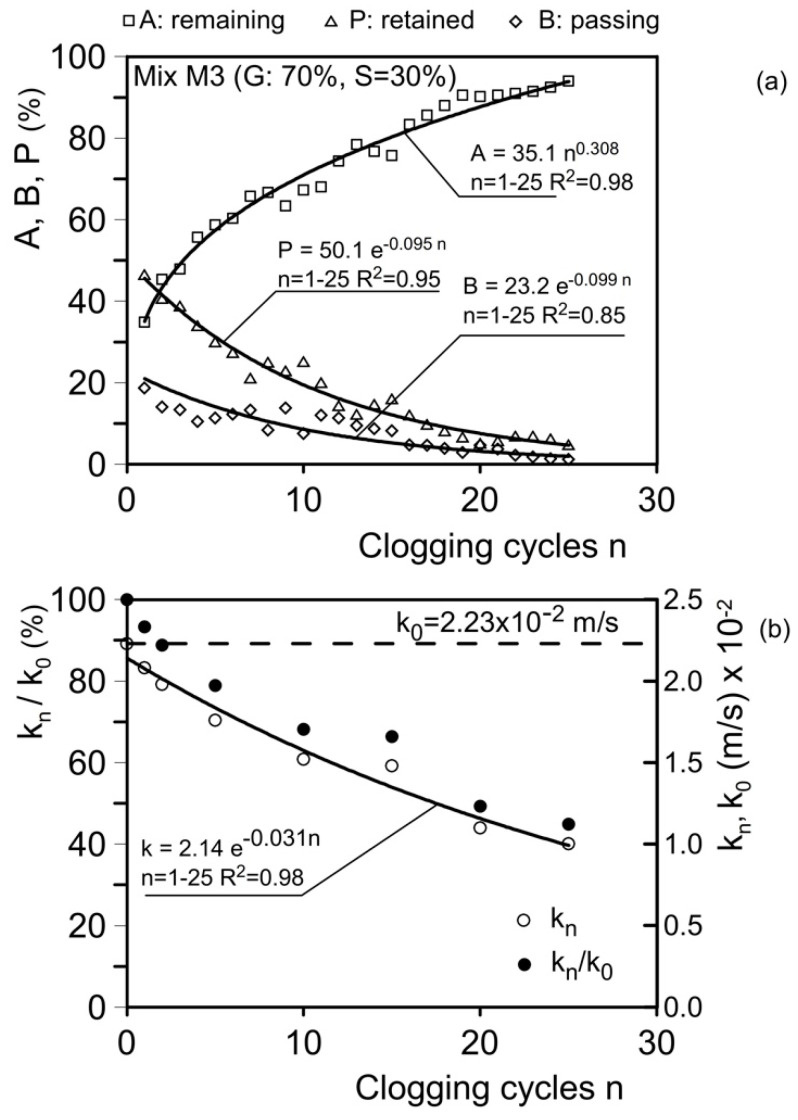
Results of clogging and permeability tests. (**a**) Material remaining at the head of the sample in weight (A), retained within the sample (P), and passing through the sample (B); (**b**) hydraulic conductivity, *k*_n_, after *n* cycles of clogging and the *k*_n_/*k*_0_ ratio (*k*_0_: unclogged hydraulic conductivity). Mix M3 (gravel G = 70%; sand S = 30%).

**Figure 17:**
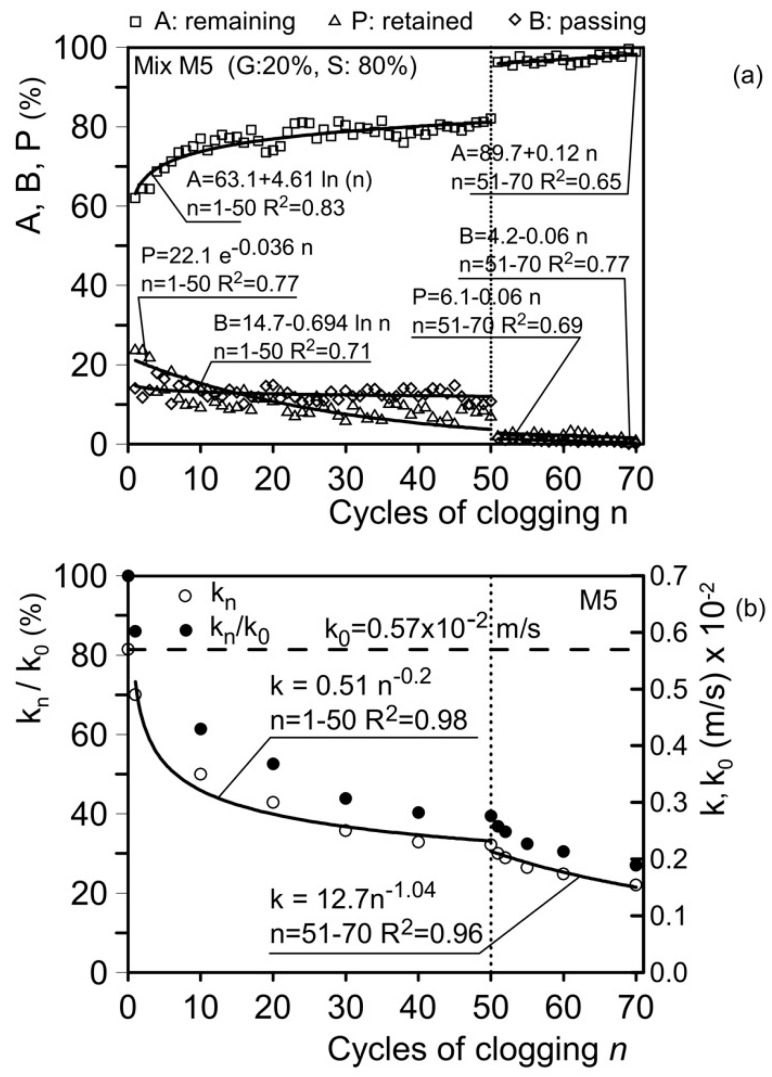
Results of clogging and permeability tests. (**a**) Material remaining at the head of the sample in weight (A), retained within the sample (P), and passing through the sample (B); (**b**) hydraulic conductivity, *k*_n_, after *n* cycles of clogging and the *k*_n_/*k*_0_ ratio (*k*_0_: unclogged hydraulic conductivity). Mix M5 (gravel G = 20%; sand S = 80%).

**Figure 18:**
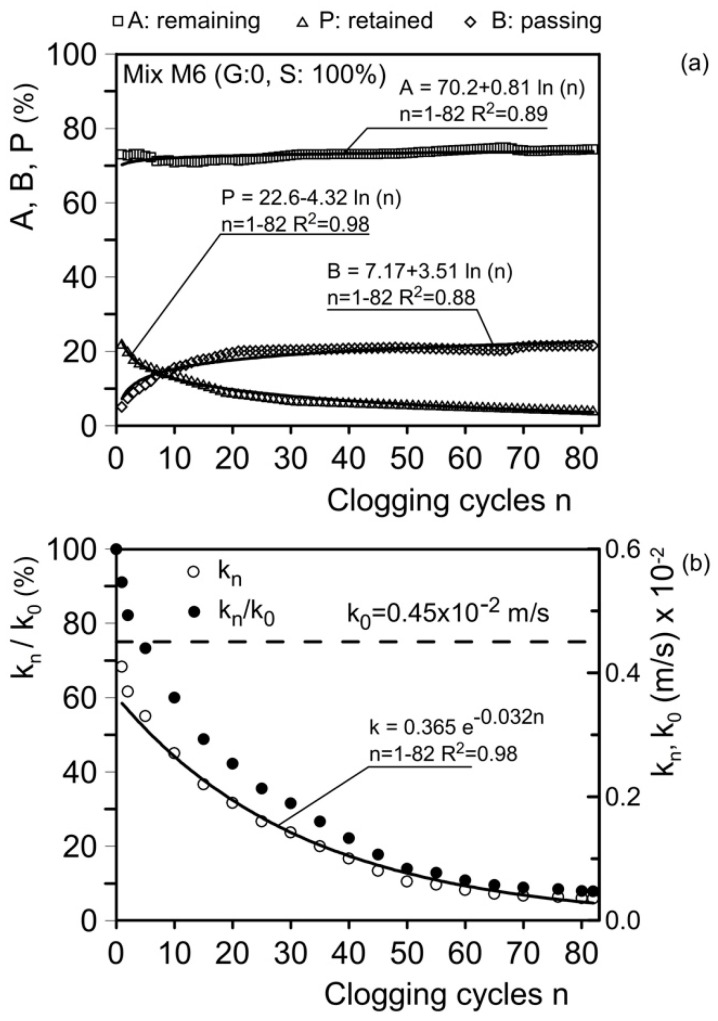
Results of clogging and permeability tests. (**a**) Material remaining at the head of the sample in weight (A), retained within the sample (P), and passing through the sample (B); (**b**) hydraulic conductivity, *k*_n_, after *n* cycles of clogging and the *k*_n_/*k*_0_ ratio (*k*_0_: unclogged hydraulic conductivity). Mix M6 (sand S = 100%).

As noted, at the end of each clogging test, the following quantities were determined: A, the material remaining at the top of the sample; B, the material passing through the sample; and P, the material retained inside the sample. The results for some of the tested mixtures are presented in Figure 15, Figure 16, Figure 17 and Figure 18.

The material remaining on the sample surface (A) and the material passing through the sample (B) were directly observed and measured (Figure 8). The material retained within the sample (P) was determined by subtracting the value (A+B) from the initial dry weight of the clogging material (W = 140 g) and was analyzed through thin sections prepared after the clogging cycles (Figure 10).

Figure 15a presents the results for mixture M1. For this mixture, the amount of material A increases with the increase in the number of clogging cycles (*n*). After the first clogging cycle, A is 13%, while P is 61% and B is 26%. During the first 20 clogging cycles (using clogging material C1, d < 75 µm), A increases to approximately 68% (*n* = 20), while P decreases to 26% and B to 14% (Figure 15a).

Fluctuations in the values of A, P, and B can be attributed to the possible removal of previously deposited particles due to filtration forces associated with the flow of the water-clogging material mixture. The relationships between A, P, and B as functions of *n* are expressed as follows (R^2^ = determination coefficient):

(2) $$A=22.78\;ln\left(n\right)-0.78\;;\;\;\;n>1;\;\;R^{2}=0.97$$

(3) $$P=70.6-15.4\;ln\left(n\right);\;\;\;\;\;n>1;\;\;R^{2}=0.93$$

(4) $$B=30.9-6.7\;ln\left(n\right);\;\;\;\;\;n>1;\;\;R^{2}=0.94$$

Figure 15b shows the hydraulic conductivity (*k*_n_) trend as a function of the number of clogging cycles (*n*). In this range of *n*, the value of *k*_n_ decreases from the initial unclogged value, *k*_0_ = 4.3 × 10⁻^2^ m/s (4.3 cm/s), to *k*_n_ = 3.84 × 10⁻^2^ m/s (3.84 cm/s) at *n* = 20.

The reduced hydraulic conductivity coefficient is associated with a decrease in pore volume due to the deposition of clogging material within the voids. In particular, this occurs due to the narrowing of effective channels and the complete occlusion of some constrictions, as schematically represented by Figure 9 and documented in Figure 10.

The relationship between *k*_n_ and *n* is expressed as follows:

(5) $$k_{n}=4.15\;n^{-0.026};\;\;\;n=1–20;\;\;R^{2}=0.98$$

Figure 15b also shows the *k*_n_/*k*_0_ ratio. For *n* = 20, this ratio is approximately 90%. For mixture M1 and clogging material C1, the reduction in permeability over a significant number of clogging cycles is not substantial. According to Equation (5), the estimated values of *k*_n_ would be 3.68 × 10⁻^2^ m/s for *n* = 100 and 3.62 × 10⁻^2^ m/s for *n* = 200. Thus, for this mixture, the residual permeability would remain approximately 80% of the initial unclogged value even after a very high number of clogging cycles.

Thus, even after a very high number of clogging cycles, the residual hydraulic conductivity for this mixture would remain close to 80% of its initial unclogged value.

These residual hydraulic conductivity values are consistent with those reported by several researchers [74,75,76,77,78,79,80,81,82,83,84,85,86,87,88,89,90]. In fact, Cai et al. (2022) [90] conducted similar experiments using pervious concrete with coarse aggregates only. They report residual permeability values ranging from 20% to 27% of the initial permeability after 35 cycles of clogging.

Clogging material C4 (d ≤ 118 µm) was used from clogging cycle 21 to cycle 82. From clogging cycle 21 to cycle 82, clogging material C4 (d ≤ 118 µm) was used. These clogging cycles were performed after five washing cycles, resulting in an initial permeability value of 3.98 × 10⁻^2^ m/s (3.98 cm/s). The first clogging cycle with material C4 corresponds to 21 clogging cycles (*n* = 21). The material remaining at the head of the sample (A) was 74.8%, the material retained within the sample (P) was 17.7%, and the material passing through the sample (B) was 7.5%.

The relationships between A, P, and B are as follows:

(6) $$A=43.8\;n^{0.18};\;\;\;n=21–82;\;\;R^{2}=0.99$$

(7) $$P=40.7\;e^{-0.33n};\;\;\;n=21–82;\;\;R^{2}=0.91$$

(8) $$B=13.7\;e^{-0.033n};\;\;\;n=21–82;\;\;R^{2}=0.99$$

The permeability, *k*_n_, reduces significantly during the first five cycles of clogging (*n* = 21–25). In fact, after the washing cycles, *k*_n_ drops from 3.98 × 10⁻^2^ m/s (3.98 cm/s) to 1.4 × 10⁻^2^ m/s (1.4 cm/s) at *n* = 25 after five clogging cycles with clogging material C4. Subsequently, *k*_n_ continues to decrease, reaching 0.46 × 10⁻^2^ m/s (0.46 cm/s) at *n* = 82 after 62 additional clogging cycles with material C4. The residual permeability is 10% of the initial unclogged value.

In this range of *n*, *k*_n_ can be described by the following equation:

(9) $$k_{n}=95\;n^{-1.264};\;\;\;n=21–83;\;\;R^{2}=0.85$$

This relationship fits the data well for *n* = 25–82, although the interpolation is less accurate for *n* = 21–24. Based on this equation, the estimated values of *k*_n_ would be 0.42 × 10⁻^2^ m/s for *n* = 100 and 0.18 × 10⁻^2^ m/s for *n* = 200.

These results confirm that the residual hydraulic conductivity remains sufficient for drainage and protecting base soils composed of fine sands.

Figure 16a presents the results for mixture M3 (gravel G = 70%; sand S = 30%). This mixture was subjected to 25 clogging cycles using clogging material C1 (d ≤ 75 µm). After the first clogging cycle, the material remaining at the head of the sample (A) is 34.8%, the material retained within the sample (P) is 46.5%, and the material passing through the sample (B) is 18.7%.

The values of A, P, and B are described by the following expressions:

(10) $$A=35.1\;n^{0.308};\;\;\;n=1–25;\;\;\;R^{2}=0.98$$

(11) $$P=50.1\;e^{-0.095n};\;\;\;n=1–25;\;\;\;R^{2}=0.95$$

(12) $$B=23.2\;e^{-0.099n};\;\;\;n=1–25;\;\;\;R^{2}=0.85$$

Figure 16b shows the trends for *k*_n_ and the *k*_n_/*k*_0_ ratio in terms of *n*. *k*_n_ decreases from its initial unclogged value, *k*_0_ = 2.23 × 10⁻^2^ m/s (2.23 cm/s), to its final value, *k*_n_ = 1.01 cm/s (1.01 × 10⁻^2^ m/s) at *n* = 25. The relationship between *k*_n_ and *n* is expressed as follows:

(13) $$k_{n}=2.14\;e^{-0.031n};\;\;\;n=1–25;\;\;R^{2}=0.98$$

This relationship predicts a value of 0.45 × 10⁻^2^ m/s (0.45 cm/s) for *n* = 50 and 0.1 × 10⁻^2^ m/s (0.1 cm/s) for *n* = 100. These data confirm that the residual hydraulic conductivity remains sufficient for drainage and protecting base soils composed of silt and coarser materials. Additionally, the filtering properties of the mixture can be classified as medium.

Figure 17a presents the results for mixture M5 (gravel G = 20%; sand S = 80%). The first 50 clogging cycles were conducted using clogging material C1. The amount of material A increases as the number of clogging cycles (*n*) rises. For *n* = 1–50, A increases from an initial value of 62% (*n* = 1) to 82% (*n* = 50), P decreases from 24% (*n* = 1) to 7.9% (*n* = 50), and B decreases from 14% (*n* = 1) to 10.1% (*n* = 50). In this range of n, A, P, and B are expressed by the following relationships:

(14) $$A=63.1+4.61\;ln\left(n\right);\;\;\;n=1–50;\;\;R^{2}=0.83$$

(15) $$P=22.1\;e^{-0.036n};\;\;\;\;\;n=1–50;\;\;R^{2}=0.77$$

(16) $$B=14.7-0.694\;ln\left(n\right);\;\;\;n=1–50;\;\;R^{2}=0.71$$

Subsequently, clogging material C2 (d ≤ 180 µm) was used. At *n* = 51, the materials register the following values: A = 96.3%, P = 2.3%, and B = 1.4%. At *n* = 70, the values are A = 98.7%, P = 1.2%, and B = 0.1%. In this range of *n*, the relationships for A, P, and B are as follows:

(17) $$A=89.7+0.12\;n;\;\;\;n=51–70;\;\;R^{2}=0.65$$

(18) $$P=6.1-0.06\;n;\;\;\;\;\;n=51–70;\;\;R^{2}=0.69$$

(19) $$B=4.2-0.06\;n;\;\;\;\;\;n=51–70;\;\;R^{2}=0.77$$

Figure 17b shows the trend of *k*_n_ in terms of the number of clogging cycles (*n*).

In this range of *n*, *k*_n_ decreases from its initial unclogged value, *k*_0_ = 0.57 cm/s (0.57 × 10⁻^2^ m/s), to *k*_n_ = 0.23 cm/s (0.23 × 10⁻^2^ m/s), representing a reduction of approximately 50%.

The relationship between *k*_n_ and *n* is expressed as follows:

(20) $$k_{n}=0.51\;n^{-0.2};\;\;\;n=1–50;\;\;R^{2}=0.98$$

For *n* = 51–70, *k*_n_ decreases from its initial value of 0.21 × 10⁻^2^ m/s (*n* = 51) to 0.155 × 10⁻^2^ m/s for *n* = 70. The relationship between *k*_n_ and *n* for this interval is expressed as follows:

(21) $$k_{n}=12.7\;n^{-1.04};\;\;\;n=51–70;\;\;R^{2}=0.96$$

This relationship predicts a *k*_n_ value equal to 0.11 × 10⁻^2^ m/s (0.11 cm/s) for *n* = 100 and 0.05 × 10⁻^2^ m/s (0.05 cm/s) for *n* = 200. These results further confirm that the residual hydraulic conductivity remains sufficient to ensure drainage and protection against internal erosion for base soils composed of fine sand, sandy silt, and silty sand.

Figure 18a presents the results for mixture M6 (sand S = 100%). This mixture was subjected to 82 clogging cycles using clogging material C1 (d ≤ 75 µm). After the first clogging cycle, the material remaining at the head of the sample (A) is 73.1%, the material retained within the sample (P) is 21.9%, and the material passing through the sample (B) is 5%. After 82 clogging cycles, A = 74.3%, P = 21.5%, and B = 4.2%. In this range of *n*, A, P, and B are expressed by the following relationships:

(22) $$A=70.2+0.81\ln\left(n\right);\;\;\;n=1–82;\;\;\;R^{2}=0.89$$

(23) $$P=22.6-4.32\ln\left(n\right);\;\;\;n=1–82;\;\;\;R^{2}=0.98$$

(24) $$B=7.17+3.51\ln\left(n\right);\;\;\;n=1–82;\;\;\;R^{2}=0.88$$

Figure 18b shows the trend of *k*_n_ and of ratio *k*_n_/*k*_0_ as a function of *n* (number of clogging cycles). The permeability coefficient *k*_n_ decreases from its initial unclogged value (*k*_0_ = 0.45 × 10⁻^2^ m/s) to *k*_n_ = 0.04 × 10⁻^2^ m/s at *n* = 82, representing a reduction greater than 90%.

The relationship between *k*_n_ and *n* is expressed as follows:

(25) $$k_{n}=0.365\;e^{-0.032n};\;\;\;n=1–82;\;\;R^{2}=0.98$$

This relationship predicts a value of 0.015 × 10⁻^2^ m/s for *n* = 100 and 0.1 × 10⁻^3^ m/s for *n* = 200. These results further confirm that the residual hydraulic conductivity remains sufficient to ensure drainage and protection for base soils composed of cohesionless silt and coarser materials. For base soils consisting of fine clayey soils, the problem of internal erosion is generally less pertinent—at least under the hydraulic gradients typically expected in geotechnical applications—due to the presence of adhesive forces between the soil particles.

These results confirm that the hydraulic conductivity of permeable concrete depends on the granulometric distribution of its aggregates, as the sizes of the voids and channels are influenced by the average grain sizes of their solid particles. In fact, permeability decreases when moving from concrete where the aggregates solely consist of gravel (mix M1) to mixtures where a progressively increasing amount of sand is added (mixes M2–M5), ultimately reaching a minimum for concrete in which the aggregate consists solely of sand (M6). Due to the physical clogging effects experienced over time, the residual permeability (long-term) of all types of tested concrete can be reduced to as low as 10–20% of its initial value. However, these hydraulic conductivity values are sufficient for draining medium–fine-graded base soils (with the hydraulic conductivity of the concrete being consistently two to three orders of magnitude higher than that of the base soils). These results also demonstrate that the filter properties of pervious concrete (protecting base soils against internal erosion) increase as the average pore sizes and constrictions of the channels decrease, thereby improving with the increasing amount of fine aggregates (sand) in the mix. Therefore, for applications of permeable concrete in geotechnical engineering where the requirement for protecting base soils against internal erosion is as important as drainage (reducing pore water pressure), the mix design must be chosen by considering the grading of the base soil and not solely the draining properties of the concrete used.

## 7. Conclusions

Based on the findings of a comprehensive experimental study on the filter properties of permeable concrete for applications in geotechnical engineering, the following conclusions can be made.

- Pervious concretes typically used for roads and other types of pavements (such as no-fines concrete) are ineffective for geotechnical applications.
- The durability and effectiveness of permeable concrete in geotechnical applications strongly depend on its filter properties, specifically, its ability to protect against internal erosion of the base soil while maintaining sufficient residual permeability (in relation to the permeability of the base soil).
- Adding sand to the aggregate mix significantly enhances the filter properties of permeable concrete for cohesionless fine-grained soils, which are the most vulnerable to internal erosion phenomena. This issue is less pertinent in clayey soils, where adhesive forces between particles help reduce internal erosion.
- Residual hydraulic conductivity decreases due to physical clogging. The reduction in permeability increases as the number of clogging cycles rises, and for a very high number of cycles, it can decrease to as low as 10% of its initial (unclogged) value. However, this residual permeability remains sufficient for draining fine cohesionless base soils.
- Additional experimentation is clearly essential to exploring possible scale effects on both the sample geometry and the piezometric gradient, particularly concerning the low gradients commonly observed in soil flow. To generalize the results and enhance the robustness of the findings, it is also necessary to employ various clogging materials, such as clayey and silty–clayey soils.
- To verify these results, it would be useful to conduct experiments in a centrifuge, as well as at a real scale and under actual field conditions.

In summary, pervious concretes designed by considering the grain size composition of the base soil exhibit residual hydraulic conductivity values sufficient to meet long-term drainage requirements, provide excellent protection against internal erosion, and possess high resistance to physical clogging (due to the gradual accumulation of fine particles eroded by subsurface water flowing toward permeable concrete drainage structures). Additionally, they offer high durability to the geotechnical systems of which they are a part.

## Figures and Tables

**Table 1:** Mean characteristics of the aggregates utilized to prepare the permeable concrete (see Figure 4). G_s_: specific gravity; γ_s_: specific unit weight.

Material	G_s_	γ_s_ (kN/m^3^)
Sand S	2.65	26
Gravel G	2.7	26.5

**Table 2:** Mean characteristics of the aggregates utilized to prepare the pervious concrete samples (see Figure 4). d_max_: maximum diameter of the particles; d_60_, d_50_, d_10_: characteristic diameters corresponding to 60%, 50%, and 10% of passing in weight, respectively; and CU: coefficient of uniformity.

Mixture	Gravel G (% in Weight)	Sand S (% in Weight)	d_max_ (mm)	d_60_ (mm)	d_50_ (mm)	d_10_ (mm)	CU (d_60_/d_10_)	Porosity n
M1	100	0	12	10	9.20	5.60	1.41	0.42
M2	80	20	12	9.11	8.31	0.92	9.90	0.40
M3	70	30	12	8.91	8.62	0.86	10.36	0.39
M4	30	70	12	1.11	1.00	0.75	1.48	0.39
M5	20	80	12	0.99	0.95	0.73	1.36	0.38
M6	0	100	1.41	0.65	0.61	0.46	1.78	0.38

**Table 3:** Mean characteristics of the water utilized to prepare the permeable concretes. Other minor components are present in negligible quantities, less than 1 mg/L.

pH	Electrical Conductivity at 20° (μS/cm)	Alkalinity (mg/L)	Sulfates (mg/L)	Potassium (mg/L)	Sodium (mg/L)	Chlorides (mg/L)	Calcium (mg/L)	Nitrates (mg/L)	Magnesium (mg/L)
7.72	100	194	161	4.6	4.6	48.5	99	5.9	2

**Table 4:** Mean characteristics of clogging materials (see Figure 5). Sands C1-C1 were obtained from VS for sieving. The specific gravity, G_s_, of the sands is 2.72, and the specific unit weight is 26.7 kN/m^3^. Materials C1–C4 were employed as clogging materials. d_max_: maximum diameter of the particles; d_60_, d_50_, d_10_: characteristic diameters corresponding to 60%, 50%, and 10% of passing in weight, respectively; and CU: coefficient of uniformity.

Clogging Material	d_max_ (μm)	d_60_ (μm)	d_50_ (μm)	d_10_ (μm)	CU
VS	2000	460	387	129	3.57
C1	75	38.1	32	7.9	4.82
C2	180	63	49	8.1	7.78
C3	600	305	251	101	3.02
C4	1180	390	322	129	3.02

**Table 5:** Mean results of the tests of clogging and hydraulic conductivity after several clogging cycles. The percentages of gravel and sand in the mixture in weight are reported in Table 2. *n*: number of clogging cycles; C.M.: clogging material. The hydraulic conductivities reported in the table refer to the minimum value of the hydraulic gradient. *k*_ini_ and *k*_fin_: initial and final values of the hydraulic conductivity coefficient, respectively.

Mixture	Clogging Material	*n*	*k*_ini1_ (m/s) × 10^−2^	*k*_fin1_ (m/s) × 10^−2^	*k*_fin1_/*k*_ini1_ (%)	Clogging material	*n*	*k*_ini2_ (m/s) × 10^−2^	*k*_fin2_ (m/s) × 10^−2^	*k*_fin2_/*k*_ini2_ (%)	*k*_fin2_/*k*_ini1_ (%)
M1	C1	1–20	4.30	3.85	89.5	C4	21–82	3.98	0.47	11.8	10.9
M2	C1	1–10	2.94	2.30	78.2	C3	11–15	2.30	1.39	60	47
M3	C1	1–25	2.23	1.01	45.3	/	/	/	/	/	/
M4	C1	1–10	1.05	0.31	29.5	C2	11–20	0.31	0.24	77.4	22.9
M5	C1	1–50	0.57	0.21	36.8	C2	51–70	0.21	0.17	80.9	29.8
M6	C1	1–82	0.45	0.03	6.7	/	/	/	/	/	/

## Data Availability

The raw data supporting the conclusions of this article will be made available by the authors on request.

## References

[1] Neville A.M., Longman Group Limited (1995). Properties of Concrete, Fourth and Final.

[2] Alam B., Javed M., Ali Q., Ahmad N., Ibrahim M. (2012). Mechanical properties of no-fines bloated slate aggregate concrete for construction application, experimental study. Int. J. Civ. Struct. Eng..

[3] Yahia A., Kabagire K.D. (2014). New approach to proportion pervious concrete. Constr. Build. Mat..

[4] Zhong R., Wille K. (2016). Compression response of normal and high strength pervious concrete. Constr. Build. Mat..

[5] Adresi M., Yamani A.R., Tabarestani M.K., Rooholamini H. (2023). A comprehensive review on pervious concrete. Constr. Build. Mat..

[6] Xiong B., Li Y., Chen B., Lu X., Gao H., Jia S. (2025). Influence of distribution of paste coating thickness on performance of pervious concrete. Constr. Build. Mat..

[7] Xiong B., Wu X., Liu W., Lu X., Gao H., Lv W. (2025). Influence of different aggregate characteristics on pervious concrete. Constr. Build. Mat..

[8] Haselbach L., Boyer M., Kevern J.T., Schaefer V.R. (2011). Cyclic heat island impacts on traditional versus pervious concrete pavement systems. Transp. Res. Rec. J. Transp. Res. Board..

[9] Liu Y., Li T., Yu L. (2020). Urban heat island mitigation and hydrology performance of innovative permeable pavement: A pilot-scale study. J. Clean. Prod..

[10] Ali M.T., Rashid M.H. (2024). The effects of coarser sand addition on thermal properties of pervious concrete. Innov. Infrastruct. Solut..

[11] Adresi M., Yamani A.R., Tabarestani M.K. (2024). Evaluating the effectiveness of innovative pervious concrete pavement system for mitigating urban heat island effects, de-icing, and de-clogging. Constr. Build. Mat..

[12] Holmes R.R., Hart M.L., Kevern J.T. (2017). Enhancing the ability of pervious concrete to remove heavy metals from stormwater. J. Sustain. Water Built Environ..

[13] Muthu M., Santhanam M., Kumar M. (2018). Pb removal in pervious concrete filter: Effects of accelerated carbonation and hydraulic retention time. Constr. Build. Mat..

[14] Shabalala A.N., Ekolu S.O., Diop S., Solomon F. (2017). Pervious concrete reactive barrier for removal of heavy metals from acid mine drainage − column study. J. Hazard. Mat..

[15] Chen X., Niu Z., Zhang H., Lu M., Zhou M., Li B. (2020). Design of a chitosan modifying alkali-activated slag pervious concrete with the function of water purification. Constr. Build. Mat..

[16] Wijeyawardana P., Nanayakkara N., Gunasekara C., Karunarathna A., Law D., Pramanik B.K. (2022). Improvement of heavy metal removal from urban runoff using modified pervious concrete. Sci. Tot. Environ..

[17] Horvath A. (2004). Construction materials and the environment. Annu. Rev. Environ. Resour..

[18] Dixit M.K., Fernández-Solís J.L., Lavy S., Culp C.H. (2010). Identification of parameters for embodied energy measurement: A literature review. Energy Build..

[19] Chandrappa A.K., Biligiri K.P. (2016). Pervious concrete as a sustainable pavement material—research findings and future prospects: A state-of-the-art review. Constr. Build. Mat..

[20] Ziccarelli M., Ferrari A., Rosone M., Ferrari A., Laloui L. (2019). The permeable concrete: A low energy consumption solution for deep draining trenches. Springer Series in Geomechanics and Geoengineering.

[21] He J., Xu S.H.S., Sang G.C., Wu Y.H., Liu S. (2024). Enhancing the Mechanical Properties and Water Permeability of Pervious Planting Concrete: A Study on Additives and Plant Growth. Materials.

[22] Zhuang P., Yan X., Wang X., Liu J. (2024). Study on the Performance Optimization of Plant-Growing Ecological Concrete. Sustainability.

[23] Ni L., Suleiman M.T., Raich A. Pervious Concrete Pile: An Innovation Ground Improvement Alternative. Proceedings of the Geo-Congress 2013: Stability and Performance of Slopes and Embankments III, ASCE.

[24] Suleiman M.T., Ni L., Raich A. (2014). Development of pervious concrete pile ground-improvement alternative and behavior under vertical loading. J. Geotech. Geoenviron. Eng..

[25] Ye G., Zhang Q., Zhang Z., Chang H. (2015). Centrifugal modeling of a composite foundation combined with soil-cement columns and prefabricated vertical drains. Soil. Found..

[26] Ni L.S., Suleiman M.T., Raich A. (2016). Behavior and soil–structure interaction of pervious concrete ground-improvement piles under lateral loading. J. Geotech. Geoenviron. Eng..

[27] Ni P., Mangalathu S., Mei G., Zhao Y. (2017). Permeable piles: An alternative to improve the performance of driven piles. Comp. Geotech..

[28] Tiwari B., Ajmera B., Maw R., Cole R., Villegas D., Palmerson P. (2017). Mechanical Properties of Lightweight Cellular Concrete for Geotechnical Applications. J. Mat. Civ. Eng..

[29] Ni P., Mangalathu S., Mei G., Zhao Y. (2018). Laboratory investigation of pore pressure dissipation in clay around permeable piles. Can. Geotech. J..

[30] Cui X., Zhang J., Chen D., Li S., Jin Q., Zheng Y., Cui S. (2018). Clogging of pervious concrete pile caused by soil piping: An approximate experimental study. Can. Geotech. J..

[31] Amran M., Onaizi A.M., Fediuk R., Danish A., Vatin N.I., Murali G., Abdelgader H.S., Mosaberpanah M.A., Cecchin D., Azevedo A. (2022). An ultra-lightweight cellular concrete for geotechnical applications—A review. Case Stud. Constr. Mat..

[32] Wang J., Zhu H.-H., Mei G.-X., Xiao T., Liu Z.-Y. (2021). Field monitoring of bearing capacity efficiency of permeable pipe pile in clayey soil: A comparative study. Measurement.

[33] Cai J., Du G., Xia H., Sun C. (2021). Comparative study on bearing characteristics of pervious concrete piles in silt and clay foundations. Geomech. Eng..

[34] Xia H., Du G., Cai J., Sun C. (2024). Model tests on the bearing capacity of pervious concrete piles in silt and sand. Geomech. Eng..

[35] Valore C., Ziccarelli M., Muscolino S.R. (2018). An experimental investigation into the permeability and filter properties of pervious concrete for deep draining trenches. Riv. Ital. Geotec..

[36] Marzulli V., Cafaro F., Ziccarelli M. (2018). Hydraulic characterization of a pervious concrete for deep draining trenches. J. Mat. Civ. Eng. ASCE.

[37] Ziccarelli M., Valore C. (2019). Hydraulic conductivity and strength of pervious concrete for deep trench drains. Geomech. Energy Environ..

[38] Ziccarelli M. (2022). Influence of Stress-level due to Self-weight on the Hydraulic Conductivity of Permeable Concrete for Geotechnical Applications. Geomech. Energy Environ..

[39] Ziccarelli M., Sapienza G., Casella A. (2024). Experimental Investigation on Shear Strength at the Permeable Concrete–Fine-Grained Soil Interface for Slope Stabilization Using Deep Socket Counterfort Drains. GeoHazards.

[40] Zhang W., Yuan Z., Li D., Zhang K., Zhao L. (2022). Mechanical and vegetation performance of porous concrete with recycled aggregate in riparian buffer area. J. Clean. Prod..

[41] Niyomukiza J.B., Eisazadeh A., Tangtermsirikul S. (2023). Recent advances in slope stabilization using porous vegetation concrete in landslide-prone regions: A review. J. Build. Eng..

[42] Niyomukiza J.B., Eisazadeh A., Tangtermsirikul S., Strauss E. (2024). Preparation of Porous Concrete Suitable for Vegetation Growth: An Approach Toward Green Infrastructure. Proceedings of the 8th International Conference on Civil Engineering ICOCE.

[43] Zeng L., Luo J.-T., Yang Z.-R., Yu H.-C., Wen W., Gao Q.-F., Zhang H.-R. (2024). Performance evaluation of vegetation concrete with carbonaceous mudstone aggregates in slope protection under wet-dry conditions. Bull. Eng. Geol. Environ..

[44] Niyomukiza J.B., Eisazadeh A., Tangtermsirikul S. (2025). Synergistic effect of calcined clay and fly ash on the performance of porous vegetation concrete. Constr. Build. Mat..

[45] Sandoval G.F.B., Inocente Jussiani E., Campos de Moura A., Casanova Andrello A., Toralles B.M. (2022). Hydraulic and morphological characterization of clogged pervious concrete (PC). Constr. Build. Mat..

[46] Barnhouse W., Srubar III W.V. (2016). Material characterization and hydraulic conductivity modeling of macroporous recycled-aggregate pervious concrete. Constr. Build. Mat..

[47] Shirke N.A., Shuler S. (2009). Cleaning porous pavements using a reverse flush process. J. Transp. Eng..

[48] Chopra M., Kakuturu S., Ballock C., Spence J., Wanielista M. (2010). Effect of rejuvenation methods on the infiltration rates of pervious concrete pavements. J. Hydrol. Eng..

[49] Hu N., Zhang J., Xia S., Han R., Dai Z., She R., Cui X., Meng B. (2020). A field performance evaluation of the periodic maintenance for pervious concrete pavement. J. Clean. Prod..

[50] Zhang X., Xu Z., Lv Q., Xu T. (2025). Hydrodynamic self-cleaning behaviors of double-layer drainage asphalt pavement. J. Clean. Prod..

[51] Terzaghi K., Paige S. (1950). Mechanism of Landslides. Application of Geology to Engineering Practice (Berkey Volume).

[52] Hutchinson J.N. (1977). Assessment of the effectiveness of corrective measures in relation to geological conditions and types of slope movement. Bull. Intern. Eng. Geol..

[53] Bromhead E.N. (1984). An analytical solution to the problem of seepage into counterfort drains. Can. Geotech. J..

[54] Stanic B. (1984). Influence of draining trenches on slope stability. J. Geotech. Eng..

[55] Iverson R.M. (2000). Landslide triggering by rain infiltration. Water Resour. Res..

[56] Rahardjo H., Hritzuk K.J., Leong E.C., Rezaur R.B. (2003). Effectiveness of horizontal drains for slope stability. Eng. Geol..

[57] Rahardjo H., Santoso V.A., Leong E.C., Ng Y.S., Hua C.J. (2011). Performance of horizontal drains in residual soil slopes. Soil. Found..

[58] Lee M.L., Ng K.Y., Huang Y.F., Li W.C. (2014). Rainfall-induced landslides in Hulu Kelang area. Nat. Hazards.

[59] Valore C., Ziccarelli M. The stabilization of a slope-viaduct system without closing traffic. Proceedings of the European Conference on Soil Mechanics and Geotechnical Engineering.

[60] Wei Z.L., Shang Y.Q., Sun H.Y., Xu H.D., Wang D.F. (2019). The effectiveness of a drainage tunnel in increasing the rainfall threshold of a deep-seated landslide. Landslides.

[61] Sitarenios P., Casini F., Askarinejad A., Springman S. (2021). Hydro-mechanical analysis of a surficial landslide triggered by artificial rainfall: The Ruedlingen field experiment. Geotechnique.

[62] Amarasinghe M.P., Kulathilaka S.A.S., Robert D.J., Zhou A., Jayathissa H.A.G. (2024). Risk assessment and management of rainfall-induced landslides in tropical regions: A review. Nat. Hazards.

[63] Di Maio C., Vassallo R. (2025). Effects of trench drain systems on pore water pressures in slow, deep, clayey landslides: Influence of hydraulic properties of the slip zone. Eng. Geol..

[64] Moraci N., Mandaglio M.C., Ielo D. (2012). A new theoretical method to evaluate the internal stability of granular soil. Can. Geotech. J..

[65] Xantakos P.P. (1994). Slurry Walls as Structural Systems.

[66] ASTM (2004). Standard test methods for maximum index density and unit weight ofsoils using a vibratory table (D4253-00 and D4254-00). 2004 Annual Book of ASTM Standards.

[67] (2002). Standard Practice for Making and Curing Concrete Test Specimens in the Laboratory.

[68] (2012). Standard Test Method for Density and Air Void Content of Pervious Concrete.

[69] (2011). Cement—Composition, Specifications and Conformity Criteria for Common Cements.

[70] Deo O., Sumanasooriya M., Neithalath N. (2010). Permeability reduction in pervious concretes due to clogging: Experimental and modeling. J. Mat. Civ. Eng..

[71] Martin W.D., Kaye N.B., Putman B.J. (2014). Impact of vertical porosity distribution on the permeability of pervious concrete. Constr. Build. Mat..

[72] West D., Kaye N.B., Putman B.J., Clark R. (2016). Quantifying the non-linear hydraulic behavior of pervious concrete. J. Test. Eval. ASTM Int..

[73] Montes F., Haselbach L.M. (2006). Measuring hydraulic conductivity in pervious concrete. Environ. Eng. Sci..

[74] Coughlin J.P., Campbell C.D., Mays D.C. (2013). Infiltration and clogging by sand and clay in a pervious concrete pavement system. J. Hydrol. Eng..

[75] Kia A., Wong H.S., Cheeseman C.R. (2017). Clogging of permeable concrete: A review. J. Environ. Manag..

[76] Zhang X., Dong L., Yu W., Ren E., Shi R. (2025). A novel approach to investigate the pore network and clogging of pervious concrete. Case Stud. Constr. Mat..

[77] Maruyama R.C., Camarini G. (2015). Properties of Cellular Concrete for Filters. Int. J. Eng. Technol..

[78] Fell R., Mac Gregor P., Stapledon D., Bell G., Foster M. (2014). Chapter 9: Design, specification and construction of filters. Geotechnical Engineering of Embankment Dams.

[79] Kevern J.T. (2015). Evaluating permeability and infiltration requirements for pervious concrete. J. Test. Eval. ASTM.

[80] Haselbach L. (2010). Potential for clay clogging of pervious concrete under extreme conditions. J. Hydr. Eng..

[81] Taylor H.F., O’Sullivan C., Sim W.W. (2016). Geometric and hydraulic void constrictions in granular media. J. Geotech. Geoenviron. Eng..

[82] Rao Y., Zhang J., Yang T., Feng J. (2021). Effect of Drying on clay clogging of pervious concrete. J. Mat. Civ. Eng..

[83] Rao Y., Fu H., Yang T., Chen H., Zhang Z., Ding H. (2022). Comparison between sand and clay clogging mechanisms of pervious concrete pavement. Sci. Rep..

[84] Sandoval B., Pieralisi G.F., Dall Bello de Souza Risson K., Campos de Moura A., Toralles B.M. (2022). Clogging phenomenon in Pervious Concrete (PC): A systematic literature review. J. Clean. Prod..

[85] Mata L.A., Leming M.L. (2012). Vertical distribution of sediments in pervious concrete pavement systems. ACI Mater. J..

[86] Fwa F., Lim E., Tan H. (2014). Comparison of Permeability and Clogging Characteristics of Porous Asphalt and Pervious Concrete Pavements Materials. Transp. Res. Rec..

[87] Walsh S.P., Rowe A., Guo Q. (2014). Laboratory scale study to quantify the effect of sediment accumulation on the hydraulic conductivity of pervious concrete. J. Irrig. Drain. Eng..

[88] Sandoval G., Galobardes I., Campos A., Toralles B.M. (2020). Assessing the phenomenon of clogging of pervious concrete (Pc): Experimental test and model proposition. J. Build. Eng..

[89] Sandoval G.F.B.B., Galobardes I., De Moura A.C., Toralles B.M., Campos A., Moura D., Toralles B.M., De Moura A.C., Toralles B.M. (2020). Hydraulic behavior variation of pervious concrete due to clogging. Case Stud. Constr. Mater..

[90] Cai J., Chen J., Shi J., Tian Q., Xu G., Du Y. (2022). A novel approach to evaluate the clogging resistance of pervious concrete. Case Stud. Constr. Mater..

